# The transition from development and disaster risk reduction to humanitarian relief: the case of Yemen during high‐intensity conflict

**DOI:** 10.1111/disa.12521

**Published:** 2022-08-01

**Authors:** Rodrigo Mena, Dorothea Hilhorst

**Affiliations:** ^1^ Assistant Professor, International Institute of Social Studies Erasmus University Rotterdam The Netherlands; ^2^ Professor, International Institute of Social Studies Erasmus University Rotterdam The Netherlands

**Keywords:** development, disaster response, disaster risk reduction, high‐intensity conflict, humanitarian aid, relief, Yemen

## Abstract

Discussions on how humanitarian aid and disaster responses can link better with development and disaster risk reduction (DRR) have occurred for decades. However, the reverse transition, from development to relief, is still poorly understood. Using the case of Yemen, this study analyses whether and how development and DRR activities adapted to the emerging humanitarian crisis when conflict escalated in the country. It concentrates on governance strategies, actors, challenges, and opportunities at the nexus of development, disaster, and humanitarian responses. Semi‐structured interviews and focus‐group discussions with aid and societal actors were conducted remotely and in Jordan. The findings show gaps in knowledge and coordination in the movement from development and DRR to relief, but also reveal spaces and opportunities to advance towards enhanced integration of action before, during, and after an emergency. This paper contributes to the literature on this nexus and critically argues for a more integrated approach to conflicts and disasters.

## نبذة مختصرة

كانت المناقشات حول كيفية ارتباط المساعدة الإنسانية والاستجابة للكوارث بشكل أفضل بالتنمية والحد من مخاطر الكوارث (DRR) منذ عقود. ومع ذلك ، فإن الانتقال العكسي ، من التنمية إلى الإغاثة ، لا يزال غير مفهوم بشكل جيد. باستخدام حالة اليمن ، تحلل هذه الدراسة ما إذا كانت أنشطة التنمية والحد من مخاطر الكوارث تتكيف مع الأزمة الإنسانية الناشئة عند تصاعد الصراع في البلاد و كيف . تركز الدراسة على استراتيجيات الحكم ،الجهات الفاعلة ،التحديات و الفرص في العلاقة بين التنمية والكوارث والاستجابات الإنسانية. تم إجراء المقابلات شبه المنظمة ومناقشات المجموعة المركزة مع الجهات الفاعلة في مجال المساعدة والمجتمع عن بُعد في الأردن. تظهر النتائج فجوات في المعرفة والتنسيق في الانتقال من التنمية والحد من مخاطر الكوارث إلى الإغاثة ، ولكنها تكشف أيضًا عن مساحات وفرص للتقدم نحو تكامل أفضل للعمل قبل وأثناء وبعد الأزمة. يساهم هذا المقال في الأدب المرتبط بهذه العلاقة ويدافع بشكل نقدي عن الحاجة إلى نهج أكثر تكاملاً للصراعات والكوارث.

الكلمات الدالة: المساعدات الإنسانية؛ تطوير؛ الحد من مخاطر الكوارث؛ رد الفعل للكارثة؛ إسعاف ؛ صراع شديد ;اليمن

## 摘要

关于人道主义援助和灾害响应如何更好地与发展和减少灾害风险联系起来的讨论已经存在了几十年。然而，人们对于从发展到救济的反向过渡仍知之甚少。本研究以也门为例，分析当该国冲突升级时，发展和减灾活动是否能够适应新出现的人道主义危机以及应该如何适应的问题。该研究侧重于发展、灾害和人道主义响应之间的关系中的治理策略、参与者、挑战和机遇。与援助和社会参与者的半结构化访谈和焦点小组讨论在约旦远程进行。调查结果显示了从发展和减灾向救济过渡的知识和协调方面所存在的差距，也揭示了在危机之前、危机期间和危机过后更好地整合行动的空间和机会。本文为有关这一关系的文献做出了贡献，并批判性地主张需要一种更综合的方法来应对冲突和灾难。

关键词： 人道援助；发展；灾难风险减少；灾害响应；救济；激烈冲突；也门

## Introduction

Places affected by high‐intensity conflict (HIC)^1^ present complex scenarios for aid interventions. In addition to low levels of effective governance, the provision of goods and services is scattered, and territorial governance is divided between multiple factions, of which the government is only one (Mena, 2018; Hilhorst et al., 2019). In many cases, crises multiply during natural hazard‐related disasters such as droughts, earthquakes, and floods, compounding the HIC. Between 1960 and 2018, an average of 67 per cent of countries were affected each year by armed conflict and these types of disaster (Caso, 2019). HIC also can foster disaster by ‘diverting national and international financial and human resources that could be used for development and for mitigation of natural hazard risk’, which may turn into a disaster (Wisner, 2012, p. 69). As a result, HIC and disasters combine to result in significant humanitarian crises, with the presence of a variety of actors providing relief.

As relief mainly focuses on the conditions of conflict, it often disregards two pertinent conditions. First, countries embroiled in HIC usually have a long history of development programmes, as they drift between periods of higher and lower levels of conflict. How to achieve a harmonious and efficient transition between humanitarian aid and development programmes has been discussed for more than 30 years by policymakers, practitioners, and academics. Ideas about linking relief, rehabilitation, and development (LRRD), the continuum, or about their nexus have dominated analyses (Macrae, 2019). This literature generally presents a movement *from* humanitarian aid *to* development; in contrast, surprisingly little work has explored the shift *from* development *to* humanitarian aid. Those deploying humanitarian aid and relief tend to ignore the prior history of development, frequently adopting a *tabula rasa* approach (Cramer, 2006; Hilhorst, 2007). Even though current resilience approaches, as we elaborate below, are more conscious of the different transitions, there is a lack of research on to what extent previous development programmes relate to, inform, or are considered by those providing humanitarian aid in times of HIC.

Second, humanitarian aid and development do not usually incorporate disaster response and prevention, as these actions are often treated as a separate domain. In reality, these processes intertwine, and disaster‐related activity can be part of both development and humanitarian interventions. Despite advances in policy recognising the importance of disaster risk reduction (DRR), humanitarian action in conflict areas tends to assume that DRR is not feasible and so focuses on relief instead (Mena and Hilhorst, 2021a). Development, DRR, reconstruction, and rehabilitation all seek to reduce people's vulnerability, recover and strengthen deteriorated livelihoods, and increase human resilience, yet remain separate in practice. Hence, this paper asks how much DRR informs disaster response, particularly in places affected by violent conflict. By scrutinising the transition from development to relief, while incorporating concerns of DRR, it contributes to insights into and reflections on the relationship among development, humanitarian aid, and disaster‐related actions during conflict, and illuminates how these fields can build on and incorporate lessons learned from past development efforts.

In Yemen, a large number of development‐related projects existed prior to 2015, many of which centred on reducing the risk of water‐related disasters. In 2015, however, most of these interventions shifted from development to humanitarian and emergency aid in response to the outbreak of civil war. Today, the ongoing conflict, compounded by droughts and floods in particular, dominates the assistance agenda. Water, sanitation, and hygiene (WASH), historically among the most critical sectors of the response in Yemen, is now seen as only one component of the broader humanitarian response (OCHA, 2018a; CARE International, 2020). Water issues are not new in Yemen: ‘years of underdevelopment, extensive damage from conflict, unstable fuel imports and natural disasters have left water and sanitation systems struggling to uphold minimum services’ (OCHA, 2018a, p. 35). Our focus on DRR, water‐related disasters, and WASH permitted an in‐depth investigation of the relations among development, prevention, and relief.

## Linking relief and preparedness in development, humanitarian aid, and disasters

Development, humanitarian aid, and disaster‐related actions have usually been treated as separate processes, and a great deal of attention has been directed at the differences between them (Frerks, Hilhorst, and Moreyra, 1999; Buchanan‐Smith and Fabbri, 2005). Even though policy may evolve and become more integrated, these distinctions continue to play a part in practice. This section reviews, therefore, how ideas of linking humanitarian aid and development have evolved historically, and how disaster‐related actions are associated with humanitarian and development ones.

### The humanitarian–development nexus

Traditionally, whereas humanitarian aid is concerned with saving lives and alleviating the suffering of crisis‐affected populations (ReliefWeb, 2008), development focuses more on medium‐ to long‐term systemic change, seeking improvements in quality of life and well‐being (Gasper, 1996). Humanitarian aid and development also differ in terms of coordination strategies, budget lines, the types of needs they seek to address, and the approaches taken to meet those needs. Importantly, development strategies typically seek to strengthen institutions and work directly through the national government, whereas humanitarian assistance centres on (international) emergency responses. The perception that humanitarian aid and development aid connect sequentially also contributed to the view that these are separate processes, blinding observers to the history of development that generally precedes moments of crisis.

Table 1 separates relief and development for analytical purposes, although, again, these two bodies of practice have increasingly become intertwined (Apthorpe, 1997; Hilhorst and Pereboom, 2015). Humanitarian actions have significant development components and, conversely, many development‐related interventions include elements traditionally seen as humanitarian aid (Frerks, Hilhorst, and Moreyra, 1999; Wood, Apthorpe, and Borton, 2001). Similarly, there is a vast assemblage of approaches building on awareness that conflict and peace are not entirely separate realities; in many countries that are neither fully in conflict nor at peace, violent conflict regularly spikes, creating a pool of important operational capacities, personnel, and local knowledge (Demmers, 2012). Moreover, disaster response can be analytically incorporated into the distinction between relief and development by distinguishing immediate response from DRR and preparedness. DRR has become engrained in international cooperation since the Hyogo Framework for Action 2005–2015 and the Sendai Framework for Disaster Risk Reduction 2015–2030, even though practice and funding streams are still uneven in their realisation of this.

**Table 1:** Ideal‐typical comparison between relief and development

Dimensions	Relief/immediate disaster response	Development/disaster risk reduction, preparedness
Objectives	Meeting immediate basic needs	Improvement of standard of living
Nature of needs	Physical, psychological	Economic, social, political
Types of intervention	Delivery of materials, provisions, and construction	Quantitative and qualitative changes in ongoing socioeconomic processes
Aid characteristics	Short term, temporary (external)	Long term (embedded)
	Incident‐related	Structural
	Relief of acute needs	Changes in vulnerability and entitlements
Management characteristics	Donor‐driven	Recipient‐focused
	Top‐down, directing	Bottom‐up, participation
Main foci	Delivery, speed, logistics, and output	Underlying processes, causalities, long‐term impact
Key context variables	Lack of infrastructure and counterparts (failed states)	Infrastructure and counterparts available
	Lack of knowledge and documentation	Knowledge and documentation available
	Media attention, fundraising	Less public attention

**Source**: authors, adapted from Frerks, Hilhorst, and Moreyra (1999).

Initial attempts to systematise ideas on how to improve the transition from humanitarian aid to development emerged in the late 1980s as LRRD. The latter sought to identify strategies to provide humanitarian relief in a way that linked well with sustainable medium‐ and long‐term development initiatives (Otto and Weingärtner, 2013; Mosel and Levine, 2014; Stevens et al., 2018).

LRRD has been criticised in three key ways for presenting a linear progression between phases (Harmer and Macrae, 2004; Hinds, 2015; Gomez and Kawaguchi, 2016). First, viewing humanitarian projects as disconnected and lacking capacity to progress to long‐term development, some have asserted that there is a gap between humanitarian aid and development that prevents a proper transition between the two domains (Otto and Weingärtner, 2013). The second contention is that the long‐term and protracted crises seen in recent decades mean that humanitarian aid has also become protracted, and that the boundaries between humanitarianism and development blur (Hilhorst, 2007; Mosel and Levine, 2014). A third argument states that the notion of LRRD views humanitarian aid as top‐down and external, although such aid could be delivered in ‘smarter’ ways (Richards, 1996), and more in sync with development, such as by building on local capacities and reinforcing people's coping abilities (Otto and Weingärtner, 2013; Hilhorst, 2018).

Supporters of ‘smart relief’ (Richards, 1996), introduced in the 1990s to make humanitarian aid more development‐oriented, draw attention to the fact that countries are rarely totally immersed in war. Pronk (1996),^2^ for example, appealed to the donor community to finance development to create ‘pockets of development’ within larger conflict‐affected contexts. Hilhorst (2007) presented a more systematic approach to smart relief with the distinction between classic and developmental relief. Developmental relief entails a preference for working through local partners and has the aim of overcoming the difference between relief and development, seeking to protect livelihoods and saving lives. These ideas later evolved into resilience humanitarianism (Hilhorst, 2018), which considers the humanitarian‐development relationship as ‘a continuous cycle where populations are constantly moving from relief to development or from development to relief in chaotic and unexpected progressions’ (Humanitarian Coalition, 2015). Another relevant contribution was the ‘contiguum’ model, reflecting the view that ‘operations in relief, rehabilitation and development may all be ongoing simultaneously within any given country’ (Commission of the European Communities, 1996, p. ii).

The previous ideas have been translated into programmes implemented in countries experiencing ‘chronic crises’. For instance, the Netherlands‐based international non‐governmental organisation (INGO) Cordaid,^3^ works with a model of ‘drought cycle management’ that aims to move interventions ‘away from [the] traditional approach of separating relief activities from development work’, positing that development agencies need to be prepared for possible stages of emergency and to plan relief measures to respond to them (IIRR, Cordaid, and Acacia Consultants, 2004, p. 41). The model has been described as the ‘accordion model’,^4^ since it depicts development and relief as needing to expand and contract depending on the context, ‘doing the right thing, at the right time’ (IIRR, Cordaid, and Acacia Consultants, 2004, p. 44). This means that interventions in these areas are as development‐oriented as possible and as relief‐oriented as necessary (Hilhorst, 2005, p. 365).

More recently, efforts to bridge the two domains have been revived, for example, in the idea of the nexus and the ‘new way of working’, a United Nations (UN) call for humanitarian and development actors to collaborate (Harmer and Macrae, 2004; ICVA, 2017; OCHA, 2017; Gómez and Kawaguchi, 2018; OCHA, 2019a; Poole and Culbert, 2019) and to integrate humanitarian and development efforts into the Sustainable Development Goals (SDGs) (OCHA, 2017, 2018b).

These approaches reveal the multidimensional nature of the linkages. First, they pertain to overcoming the separation of activities and phases through time. Second, they require coordination across different types of agencies and agendas, such as the SDGs. Third, they call for more flexibility in programming. Fourth, they aim for nuanced geographical consideration, with different approaches applied in different parts of a country.

### Advancing the nexus

Overcoming the divide between development and humanitarianism has thus been on the agenda for quite some time. Apart from the LRRD discussions elaborated above, increasing attention is now being paid to the triple nexus, resilience approaches, and human security.

The triple nexus concerns linkages between peace (peacebuilding), humanitarian aid, and development. It arose from recognition that development, peace, and stability happen in non‐linear and context‐specific ways and, importantly, that communities do not have isolated or one‐dimensional needs (IASC, 2020). In addition, this approach explores how peacebuilding can inform or bridge the transition from development to humanitarian aid (Ministerie van Buitenlandse Zaken, 1993; Slim, 2017). Similarly, peacebuilding also seeks to advance early warning indicators of conflict to facilitate early responses to a crisis (van de Goor and Verstegen, 1999); and many approaches acknowledge that conflict and peace are not entirely separate realities (Frerks, 2013; IASC, 2020).

Resilience is a concept that was discussed as long ago as the 1960s, but which has been a solid component of agendas since 2008 (Otto and Weingärtner, 2013). Depicted as a broader concept than LRRD, resilience can bring together different sectors, people, and agendas beyond humanitarian and development ones (Otto and Weingärtner, 2013; Mosel and Levine, 2014). The European Commission (2013, p. 3) has advocated resilience‐building on the grounds that ‘[a]ligning humanitarian and development aid to national resilience strategies and frameworks is a precondition for sustainable results’. This is consistent with Oxfam's (2013, p. 28) invitation to donors, the UN, and INGOs to break down institutional barriers and ‘work across the humanitarian–development divide, strategically linking or integrating humanitarian and development work’. The main idea behind resilience is that fostering people's capacities during crises can reduce the need for emergency relief while creating long‐term opportunities to resist and recover from shocks (Otto and Weingärtner, 2013; Mosel and Levine, 2014). However, as Mosel and Levine (2014, p. 5) emphasise, ‘[r]esilience *in* crises, as opposed to resilience to crises, is not yet high enough on the agenda’.

Lastly, the human security approach can also generate relevant insights. In a variety of scenarios, paying attention to ‘vulnerabilities, risks, and forces of disruption and destruction’ (Gasper and Gómez, 2014, p. 2) allows for the study of the evolution of crises and how they are (or are not) addressed in terms of the development–humanitarian continuum. Many policies have progressed, yet there is little empirical evidence on how the transition from development to relief is dealt with in practice, and crisis response rarely seeks to build explicitly on previous development efforts.

### From DRR to disaster response

For several decades, the dominant method of modelling disaster action has been in reference to the disaster cycle, a comprehensive approach that includes the ‘sum total of all activities, programmes and measures which can be taken up before, during and after a disaster with the purpose to avoid a disaster, reduce its impact or recover from its losses’ (Vasilescu, Khan, and Khan, 2008, p. 44). The thinking behind the cycle is that after responding to a disaster, processes of reconstruction and rehabilitation occur in ways that reduce the risk of disasters in the future (Wisner, Gaillard, and Kelman, 2012). These processes and all activities to learn, anticipate, mitigate, and prepare for a forthcoming disaster are part of the broader DRR approach. This has been described as a strategy to prevent disasters *and* a component of the response to them, since the ways in which a disaster is addressed can affect the potential for recurrence of a disaster and the creation of new ones (Blaikie et al., 1994; UNDRR, 2019).

Disaster governance goes beyond the cycle and incorporates the management of and responsibilities for disasters, including DRR, disaster response, disaster knowledge production, and related policies and normative frameworks, with multiple actors focusing on social, economic, and political dimensions (Tierney, 2012; Field and Kelman, 2018; Hilhorst et al., 2019; UNDRR, 2022). Disaster governance and actions, therefore, integrate efforts to reduce vulnerability into relief efforts to save lives, combining work that can be seen as part of development and humanitarian assistance.

DRR initiatives are expected to prevent disasters or to prepare societies to respond better to them. One example of this is early warning mechanisms, which are implemented during the prevention stages and can significantly reduce the impact and risk of disasters. For instance, in the ‘seamless’ approach of the Japan International Cooperation Agency (JICA, 2015), a more prepared response comes from early stage prediction of the occurrence of a disaster, timely dissemination of warnings, effective alerting or evacuating of residents, and immediate relief provision to affected areas when a disaster occur. Another DRR strategy that informs and supports disaster response is capacity strengthening or sharing—specifically, non‐structural capacity development, such as training community‐level first responders to help people living in disaster‐prone areas to respond adequately in the aftermath of a disaster. Despite more DRR in development, ODA (official development assistance) donors are still not allocating significant components of their budget to DRR (Sparks, 2012). Our own calculations show that between 2016 and 2019, for instance, only 0.7 per cent of total ODA‐committed funds were for disaster prevention and preparedness.^5^ While recognising the complications of advancing the agenda of DRR in development, we nonetheless bring DRR into the analysis of development because both can be similarly marked by disruptions to activities, coordination, and flexible programming amidst conditions of escalating conflict.

One setback with capacity sharing and early warning mechanisms, particularly in places affected by HIC, is that they assume the presence of governmental structures that can initiate and coordinate these endeavours. The Sendai Framework states that ‘[d]isaster risk reduction requires that responsibilities be shared by central Governments and relevant national authorities, sectors and stakeholders, as appropriate to their national circumstances and systems of governance’ (UNISDR, 2015, p. 13). However, in HIC scenarios, national and local governance structures are significantly fractured, resulting in dependency on the promotion and coordination of disaster‐related actions by international actors (Macrae, 2019; Mena, Hilhorst, and Peters, 2019; Mena and Hilhorst, 2021a).

**Figure 1:**
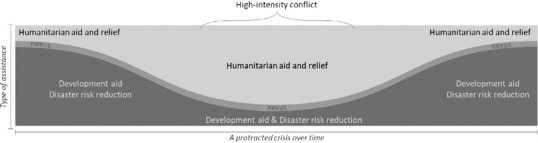
Integration of development, prevention, and relief **Source**: authors.

Transitioning from development and DRR to relief and creating a better link to previous development efforts are especially important in the context of disasters (Macrae, 2019). This movement, however, needs to overcome the sequential and linear approach to it (owing to the critiques presented above), and one way of doing so is to see the process as a *balancing act*. This idea aligns with the resilience, triple nexus, and LRRD approaches, which all acknowledge that at times, development will be more significant, whereas at other points, more humanitarian aid will be needed, yet both types of assistance are essential for dealing with disasters and conflicts and for supporting peace and stability. Figure 1 proposes a way to illustrate this balancing act between the types of assistance during times of prolonged crisis, and how different policy approaches increasingly seek to integrate flexibly development, DRR, and humanitarian action. This paper explores how the transition between development and relief takes shape in an actual situation of conflict escalation, that of Yemen.

## Research questions and methods

Based on the arguments above, we used the case of Yemen to investigate the transition from development to humanitarian aid in a HIC setting affected by disasters. Questions guiding the research process were:

- What happened to the development and DRR actors working in this context and what happened to their programmes after the conflict erupted?
- Were these actors able to remain in Yemen, and have they found alternative ways of working in the country?
- Were the programmes able to continue with modifications, or were they cancelled, paused, or completely overhauled?
- What scenarios did humanitarian actors find when they responded to the crisis and disasters?
- Which coordination mechanisms were in place for the shift from development to humanitarian aid actors and actions?

To answer these questions, we designed a qualitative case study comprising both remote research and fieldwork phases. While the project had a special focus on WASH programmes and water‐related disasters, it aimed to learn more broadly about disaster and the general transition process from development to relief.

After an extensive literature review, the first author conducted fieldwork in Jordan in November and December 2019. The impossibility of gaining access to Yemen drove the decision to use remote research techniques, mostly from Amman, Jordan, the main city used to fly into and out of Yemen by international and Yemeni aid, development, and societal actors. This made it possible to interview people during flight layovers and between meetings. Jordan also hosts the offices of multiple non‐governmental organisations (NGOs), donors, UN agencies, and other organisations working in or on Yemen. In cases where it was not possible to reach an individual by other means, interviews were conducted using video conferencing. These remote interviews lasted for one hour on average. Table 2 provides an overview of the participants in the research.^6^

**Table 2:** In‐depth interview and focus‐group participants

Participant type	Number	Description
Yemeni NGO representatives	7	Managers, country directors, and staff members of local and national NGOs
International NGO representatives	6	Managers, country directors, and staff members
UN managers and staff	5	Programme managers and directors in Yemen and at the international level
Internationally recognised government representatives	2	Yemen's internationally recognised government
Donors	5	Interviews with representatives of two national donors and one intergovernmental donor
Academics	3	Academics conducting research about and in Yemen
Private sector actors	2	Private sector actors associated with aid provision
**Total**	**30**	

**Source**: authors.

We also explored the possibility of working with a local researcher. However, after talking to key informants and other researchers in Yemen, it was determined that local researchers would be exposed to multiple risks. The research design and questions were revised accordingly, so that they could be answered via remote research. The main change was to the level of our analysis. Local perspectives were difficult to garner, leading our analysis to focus more on international development and humanitarian assistance efforts. Despite this limitation, we were able to include in our study Yemeni NGOs (YNGOs), government officials, academics, and the private sector in Yemen. Another modification involved the use of a disaster lens to concentrate more on the general transition process from development to relief, producing a more detailed account of the WASH sector and water‐related disasters.

For data analysis, we used thematic analysis techniques informed by three predetermined main themes:

- development and DRR initiatives before and during the crisis;
- coordination and transition strategies and processes; and
- individual actors and organisations before and during the crisis.

## The case of Yemen: conflict, disaster, and a major humanitarian crisis

Civil war broke out between the Houthis, members of an Islamic political movement, and Yemen's internationally recognised government in the second half of 2014 (Edwards, 2019), leading to the collapse of essential services and institutions and a state of fragile governance and socioeconomic catastrophe (World Bank, UNCT, and European Union, 2015; OCHA, 2018a). The situation was described as the ‘largest humanitarian crisis’ worldwide in 2017 and 2018 (Al Jazeera, 2017; United Nations, 2018). By 2019, more than 24 million people (three‐quarters of the population) were in need of humanitarian assistance and 3.4 million people were internally displaced (OCHA, 2018a; IMDC, 2019; UNHCR, 2019). As a result, aid agencies launched an appeal for more than USD 4.1 billion.

Adding to the social crisis, the *National Report on Disaster Risk Reduction* notes that Yemen is also vulnerable to hazards such as ‘flash floods, earthquakes, technological hazards, civil conflict, population growth, urban migration, extreme climate events, desertification, soil erosion, landslide, mudflow, locust invasions, depletion of groundwater aquifers and disease epidemics’ (Ministry of Water and Environment, 2005, p. 1). Yemen was previously ranked among the most disaster‐prone countries in the Middle East and North Africa region (World Bank, 2014). However, specific up‐to‐date information about disasters affecting the country and detailed historical data are scattered. The Emergency Events Database (EM‐DAT)^7^ provides one of the few statistical accounts of Yemen's disaster history, but it lacks information on droughts in 2018 and 2019 (see Table 3).

**Table 3:** Number of casualties and people affected by disasters in Yemen (1900–2019)

Disaster type	Total number of deaths	Total number of people affected by disasters
Bacterial disease	759	462,020
Riverine flood	596	347,839
Tropical cyclone	75	140,939
Flash flood	274	137,678
Earthquake	10	40,039
Flood	64	23,458
Viral disease	35	3,494
Landslide	96	31
Volcanic ash fall	6	15
Storm	30	0
Drought∗	–	–

**Note**: ∗ EM‐DAT does not provide statistical accounts of droughts in Yemen in 2018 and 2019.

**Source**: authors, using information from EM‐DAT (downloaded in December 2019).

Water‐related disasters (that is, droughts, floods, and landslides), including water‐borne (bacterial) diseases such as cholera, have had the greatest impact on Yemen in terms of casualties and economic losses (PreventionWeb, 2014; World Bank, 2014; GFDRR, 2015). The causes of drought in Yemen, such as rampant groundwater exploitation and ‘a framework that has promoted expansion rather than efficient use and sustainable management’ (2015, p. 251), are shared with other Middle Eastern countries, but Yemen, in particular, is considered to be ‘one of the most water‐scarce countries in the world’ (Varisco, 2019, p. 1). In addition, as noted by Weiss (2015, p. 252), ‘the depletion of Yemen's aquifers is especially problematic since Yemen has no perennial rivers and is forced to rely for its daily water needs on groundwater and other sources of water that ebb and flow according to the season’.

Drought also induces local conflicts in the country, particularly over the control of groundwater sources (Weiss, 2015), and plays a role in internal migration (Ismail, 2009). As a consequence of the water crisis, including a lack of access to drinkable water, reduced or failed crop production, and land degradation, food insecurity in Yemen has reached severe levels, intensifying the risk of famine (FAO, 2018; OCHA, 2018a; IPC, 2019). Heavy rainfall and flash floods have also affected humanitarian aid and relief provision, as these events have ‘damaged shelters, clinics, child friendly spaces and classrooms, and spoiled stocks of food rations and hygiene kits, and flooded WASH facilities’ (OCHA, 2019b, p. 2). As stated by a UN climate change specialist, not only has the war affected the country, ‘[b]ut climate change and disasters have been an important “threat multiplier” over many years, exacerbating food insecurity, decimating water reserves, expanding drylands and creating underlying levels of social vulnerability’ (Walid, 2017, p. 1).

## Findings

This section presents the results on the transition from development to humanitarian aid in Yemen, with a focus on water‐related disasters. It includes findings concerning which actors were present, their coordination mechanisms, and the objectives of development and humanitarian aid in the country before and during the crisis. It also highlights important limitations of knowledge and challenges that prevent better coordination and enhanced movement from development to humanitarian relief.

### The turn from development to humanitarian aid for water‐related disaster management

To understand the transition from development to humanitarian aid using a disaster lens, it is necessary to clarify how and by whom disaster issues were addressed prior to and during the crisis. Both the interviews and the literature review showed that beforehand, most programmes in Yemen working to reduce the risk of water‐related disasters were part of general WASH projects focusing on access to sanitation services in urban areas (Moore and Fisher, 2012; World Bank, 2017; Abu‐Lohom et al., 2018). These development programmes were actively organised or funded by the Food and Agriculture Organization of the United Nations (FAO), the World Food Programme (WFP), the World Bank, the United Nations Development Programme (UNDP), and NGOs, working in partnership with the Government of Yemen, local organisations, and private companies (GFDRR, 2015).

The few DRR activities that have been implemented also aligned with development schemes and included rural areas and the agricultural sector. For instance, in 2009, multiple projects seeking to address drought and floods were developed by FAO and WFP in partnership with Yemen's Ministry of Agriculture and Irrigation and local authorities. The programmes included the delivery of subsidised seeds, educational components, and initiatives to introduce integrated water resources management practices. As noted by WFP's Deputy Regional Director for the Middle East, Central Asia, and Eastern Europe, Philip Ward, these programmes were ‘designed to support a developmental process rather than a dependency on food aid’ (Ismail, 2009).

Since mid‐2014, the World Bank (2017, p. ix) pointed out, ‘not only have advances in WASH provisions made over the last decade been halted but also the country has experienced wholesale physical destruction, institutional degradation, and movement of internally displaced people (IDPs) that have contributed to an alarming deterioration in WASH service’. Similarly, the GFDRR's (2015) *Country Profile* stated that the country's disaster risk management plan, updated and ratified in 2010, was suspended due to political unrest. Consequently, from 2014, water issues have transitioned from being seen as part of long‐term development to being addressed and framed as part of the ‘general humanitarian response’, as expressed by an INGO member of staff. Regarding drought and water scarcity, after the onset of the crisis, the solution was delivering water by tanker trucks (YNGO, UN, and INGO interviews; see also Whitehead, 2015). A former Yemeni government official working on water‐related programmes mentioned in an interview that water scarcity measures revealed a problematic emergency mentality, as ‘they [UN agencies and INGOs] just give water to people without thinking that there might [be] better solutions. This can create more problems.‘

Hence, if water problems were addressed before the crisis by organisations developing infrastructure for water access, it is valid and important to ask what happened to these programmes and the implementing actors. Most interviewees responded to this question by saying ‘the crisis happened’ and underlined that Yemen is now a conflict‐affected place with new actors involved in tackling water‐related disasters.

### Actors, coordination, and information before, during, and after the transition from relief to development

**Figure 2:**
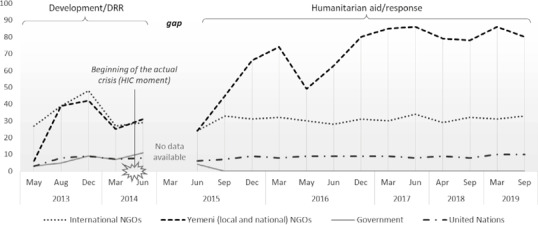
Numbers of organisations operating in Yemen, 2013–19 **Source**: authors, using information derived from *Yemen: Monthly Organizations Presence Who, What, and Where (3Ws)* reports published by the United Nations Office for the Coordination of Humanitarian Affairs (https://www.humanitarianresponse.info/operations/yemen (2013–18) and https://reliefweb.int/ (2018 and 2019)). All reports downloaded in December 2019.

A similar number of INGOs and YNGOs were working in the country in 2013 and 2014, supported by multiple UN agencies, while a growing number of governmental institutions were acting as partners and implementers (see Figure 2). Many of these organisations were addressing water‐related disaster risk (interviewees from YNGO, UN, and two donors). However, in June 2015, because of the escalating conflict, the scenario changed radically and the number of YNGOs started to mount, doubling between 2017 and 2019 (as seen in Figure 2).^8^

Two main explanations for the rising number of YNGOs emerged from documents and interviews. First, during the transition from development to humanitarian aid from late 2014 to early 2015, many INGOs continued to work in the country but with minimal staff, implementing projects via partnerships with YNGOs. Second, the crisis offered a business opportunity for local and national actors in the sense that it created new needs and left some demands unmet by development actors or INGOs. This second point was also mentioned in a focus group with YNGO representatives, in which some participants also shared how they participated in the formation of new YNGOs or the rebranding of old ones, to apply for funds and tackle the ‘new’ needs. Many stressed that multiple ‘old’ problems (from before the current civil war), such as water‐related disasters, were still present and even worsening in Yemen, but the aid sector was not concentrating on them anymore or it was doing so using a different strategy, including the delivery of water by tanker trucks.

Figure 2 illustrates a gap in information in terms of which organisations were present and working in Yemen from August 2014 to June 2015. Little is known about which activities and programmes were implemented in Yemen during these months. The interviewees from all sectors noted that, when the civil war started, many organisations stopped or paused their interventions, only restarting certain activities after July 2015. A Yemeni staff member of an INGO stated: ‘Almost everyone left; the foreigners out of the country and most of us to our houses. There was no job; the projects stopped’. A *Situation Report* produced by the United Nations Office for the Coordination of Humanitarian Affairs (OCHA) pointed out that ‘[m]any international humanitarian staff have been temporarily relocated outside of Yemen due to growing insecurity. Humanitarian operations continue to be implemented and coordinated in‐country with remaining international and national staff’ (OCHA, 2015, p. 6). Activities were carried out on a smaller scale, with many programmes assuming a ‘skeleton operation’, maintaining core activities and minimising the presence of personnel.

When we asked the interviewees about this, most indicated that it was because they were in a HIC setting; levels of violence and avoiding putting people at risk were the main reasons given. They mentioned, too, the impossibility of implementing projects, considering that they would not be sustainable, and that project participants would be displaced. INGOs and UN interviewees added that before the crisis, most of the projects were implemented jointly with civil actors and the government, but that was not possible during a HIC. Expanding on the previous point, they said that development‐related activities, including preventing the risk of water‐related disasters, are tasks that involve the government. This idea echoes the call in the Sendai Framework regarding the role of governments in disaster‐related actions and the challenges posed during a HIC.

Development‐related organisations, including donors, worked remotely to continue operations in Yemen, described as ‘resilience programmes’ by donors and UN actors. They tried not to transition fully to humanitarian relief, instead carrying out interim projects to, in the words of a UN development manager, ‘try to help beyond just saving lives, but … [they are] not [as] sustainable as we would like them to be’.

This gap in knowledge is also illustrated by the United Nations Information Centres, which started publishing a monthly newsletter detailing UN activities in Yemen in 2014, but paused all news production from May 2015 to May 2018,^9^ and by OCHA, whose country reports were interrupted from October 2014 to March 2015. The latter generally came out monthly before the crisis, but they have been less regular since resuming production in March 2015.

Not surprisingly, the transition from more developmental activities to those aimed at relief was described as uncoordinated. A ‘Consultative Meeting for Yemen’, which took place in Larnaca, Cyprus, on 6–8 October 2015, concluded that ‘[t]here is a need for better coordination across all actors’ (World Bank et al., 2015, p. 13).

Before the crisis, development activities were mainly coordinated by UNDP, as the UN Resident Coordinator (RC) was also the Resident Representative of UNDP (United Nations, 2015). General aid governance was organised under the Cluster System; however, as one interviewee put it, ‘[b]efore 2015, the clusters were there, but there was no coordination between them. The main activity for [the] clusters was to avoid any duplication of projects.‘

As humanitarian needs were also present in the country, working closely with the RC was the Humanitarian Coordinator (HC) for Yemen. The HC ‘coordinates the urgent and lifesaving assistance in the country [… and] collaborate[s] closely with the Yemeni people, the Government and other International and Yemeni partners, to ensure that the support of the United Nations in Yemen best serves to realize the future Yemenis want’ (United Nations, 2014, p. 2).

This statement shows that although the focus in Yemen was on development‐related activities, some international humanitarian actors were present, such as OCHA, which had been in the country since 2010, primarily to support the HC. This reinforces our main research question on why the transition and coordination presented important gaps, especially knowing that there was a humanitarian actor in Yemen. In a second round of interviews, UN and INGO participants said that the fundamental reason is that although there were humanitarian institutions, their mandate was to support the HC and the people in those positions had expertise in doing so. It is for the same reason that the governance of the response changed in 2016, when a newly appointed RC also assumed the role of HC, and made strengthening humanitarian aid the primary assistance objective in the country.

From a societal coordination and governance perspective, although all interviewees mentioned the authority of the Houthis and the internationally recognised government in their territories, they also described this duality of power as a significant issue. The two entities had limited governance capacity and differing (and sometimes unknown) agendas and procedures. This added a north–south geographical divide and a coordination challenge to the problematic division between development and humanitarian aid.

### Objectives, main foci, and types of interventions

Two crucial dimensions shown in Table 1 are objectives and main foci. Although some NGOs and other organisations working on water‐related disasters before the crisis were able to continue to address the same topics as part of WASH schemes during the HIC, they mentioned having to change their focus when funding and support from donors and other organisations were no longer available. As a YNGO manager stated: ‘We tried to keep doing it [development‐related WASH programmes], but the problem is that donors do not support us anymore, so we had to change to emergency [such as to food assistance]‘.

An in‐depth analysis of the *Yemen: Monthly Organizations Presence Who, What, and Where (3Ws)* reports published by OCHA^10^ (see Figure 2) confirmed this situation. In May and June 2016, 14 YNGOs working on early recovery, protection, WASH, or education ceased operations, and 14 new YNGOs emerged in the following month, working on shelter, non‐food items, and camp coordination and management. Similarly, about one‐half of the development actors interviewed cited funding and donor agendas as the principal reasons for cancelling development programmes. Whereas YNGOs described how funds were cancelled or money ‘stopped flowing’, INGOs and UN agencies reported having to return donor funding. Most donors highlighted that the priority in Yemen was responding to the humanitarian crisis and that insufficient resources and capacities made it unfeasible and unsustainable to pursue other non‐emergency initiatives. In comparison with other HICs around the world, the interviewed donors noted that the same dilemma always materialised: HIC scenarios present levels of humanitarian need that mean that other priorities have to be postponed until lower levels of conflict are present.

An important nuance here is that, although there were reduced programmes with development aims during the crisis, many YNGOs continued to conduct development‐like activities by adapting or disguising these projects. For instance, a WASH project financed by humanitarian aid funds from the UN's Country‐based Pooled Funds scheme^11^ sought to improve water treatment to reduce cholera and other water‐related diseases. While this part of the project was not included in the formal log frame or officially reported by the implementing NGOs, some project funds were used for educational activities associated with personal hygiene and reproductive health. An aid actor involved in the project explained why they carried out these endeavours without support from donors or government authorities: ‘We had experience [of] working on these topics in the past, and we know they are important for long‐term and forever problems’. When asked about projects centred around water‐related disasters, the research participants said that the vast majority of them are based on emergency solutions, in part because long‐term recovery and DRR work ‘require that you know much more [in comparison with the logistics of delivering water by truck], for example, about agricultural things, water management, and climate things’ (INGO interviewee). Similar ideas were reinforced in a focus group with YNGO participants.

Limited knowledge to guide the transition and to manage different interventions

Transitioning from development to relief meant a change in the type of interventions conducted, requiring the managerial knowledge and capacities necessary to carry out the new activities. Despite the presence of the development organisations that remained in Yemen (albeit with minimal capacity), the humanitarian actors newly active in the country were unable to manage their actions in a way that reflected learning from or connected with development projects. A UN manager emphasised:

*It would be great to arrive and have someone inform me about all that is happening on the ground and how we connect with those efforts. UNDP and some people I know here helped me a lot, but there is a lot left out of what they do or managed*.

Besides UNDP, other actors expected to know about development initiatives are the Government of Yemen and the HC. However, the research participants were notably silent regarding their knowledge of the roles of the HC and OCHA before the crisis, and even more so about past activities in relation to water‐related disasters. This highlights the lack of information and knowledge transferred before and during the crisis. In combination with Yemen's fractured governance, this lack of knowledge indicates a failure to connect with previous projects, as well as insufficient awareness of the continuation of projects and the implementation of new ones.

Another relevant gap, mostly mentioned by YNGO and government representatives, involved a lack of knowledge of managing large‐scale humanitarian aid interventions and the loss of on‐the‐ground development expertise. In the interviews, local and national NGO representatives expressed two main ideas. First, although they lacked capacity in and knowledge of how to run large‐scale humanitarian projects, no other work was available for them. This matter was presented as more acute in relation to addressing water‐related disasters in HIC scenarios because, according to interviewees, it is an area that requires specialised knowledge. Second, these actors had expertise in development‐related interventions in times of crisis, but their experience of these programmes was lost because donors did not allow them to integrate this approach, or they did not have the necessary resources or time to do so. A Yemeni INGO manager explained: ‘We didn't know what to do. It's similar to what I was doing, so I could improvise, but it was not the same’. This kind of improvisation was referred to multiple times by different actors, who underscored that they had to respond using their experience of development.

The claim about the underutilisation of development‐related expertise has two caveats. First, many aid actors said that they included some development elements in their emergency interventions. They drew on their experiences, knowing ‘that implementing the project as it is mandated will only bring short‐term solutions’, in the words of one YNGO manager. These changes were sometimes discussed with and approved by donors, but usually were not formalised, as was the case in the example of the informal education element of the WASH project presented above. Implementing projects in this way was also seen as a strategy to work in conflict‐affected settings because many development actions were considered to be in line with the agenda of one of the warring parties. Second, multiple interviewees working in the country mentioned that development interventions preceded the start of ‘official development’: ‘Development starts earlier in the country than from outside’ (YNGO manager). One reason for this is that, in many places, the macro conflict did not directly affect the local population, leaving spaces (or ‘pockets of development’) open for the implementation of development projects. Conversely, before the conflict intensified, ‘pockets of emergency’ in Yemen were already receiving humanitarian aid (OCHA, 2014).

### Challenges and ways of moving forward

The development–humanitarian transition presents multiple challenges and opportunities, many of which are similar to the obstacles facing the transition from humanitarian aid to development (Macrae et al., 1997; Otto and Weingärtner, 2013; Hinds, 2015). For instance, for both humanitarian aid and development actors, naming organisations with the expertise or knowledge to work in both domains was difficult, meaning that there were no organisations to act as bridges during such a transition. This is an interesting finding given that most organisations have multiple mandates: they have a humanitarian mandate and one or more other mandates, such as development or peacebuilding (Hilhorst and Pereboom, 2015). However, as noted, interviewees pointed out that the organisations can be multi‐mandate, but specific projects or their individual expertise are not. Similarly, these organisations often work with different mandates and even under different funding schemes, which present the challenge of finding a mechanism for evaluating programmes during protracted transitions—recognised as an issue in LRRD as well (Buchanan‐Smith and Fabbri, 2005; Otto and Weingärtner, 2013).

The case of Yemen also demonstrates the complexity of linking interventions across different regions of a country affected by a HIC, especially if they have different needs. This relates to the LRRD literature on the sustainability problem of implementing long‐term development projects among populations that may return to their hometowns during the recovery phase (Buchanan‐Smith and Fabbri, 2005; Otto and Weingärtner, 2013; Mosel and Levine, 2014). Some research participants expressed the opposite concern: that trying to enhance the link between development and humanitarian aid might lead to investing in programmes and projects that would have to be cancelled because of the conflict, which is also a concern of triple nexus initiatives (CIC, 2019). LRRD and the development–humanitarian nexus also share ‘the two‐world challenge’ of finding commonalities and coordinating despite different mandates, principles, partner strategies, imperatives, languages, speeds, time frames, and funding mechanisms.

Another major difficulty pertains to the fractured governance systems in HIC contexts, resulting in the absence of bodies to manage the transition from development to humanitarian aid. Many of the interviewees pointed to the lack of coordination between departments with different mandates within a single organisation. Furthermore, the budget and funds for humanitarian aid in Yemen were perceived to be, in the words of one INGO representative, ‘too much and unmanageable’. In relation to the transition from development to relief, this presents accountability‐related issues, particularly when large amounts of funding come from multiple sources.

The challenges shared by LRRD and the development–humanitarian nexus also suggest opportunities. For example, learning how to overcome ‘the two‐worlds’ problem will benefit both approaches and provide an opening to join efforts centred on this task. The continued presence of many organisations working on development in Yemen during the crisis can be seen as a chance to design better transition strategies within each organisation, which some interviewees described as easier to implement than such efforts between organisations. The existence of ‘pockets of development’ raises the possibility for the development–humanitarian nexus to be a process in which the two domains of assistance complement each other during various moments of the crisis (as Figure 1 illustrates).

The case of Yemen, aligning with the research participants' statements, also suggests that the existence of ‘pockets of (conflict‐related) emergency’ may play an important role in moving the development–humanitarian nexus forward. First, this situation may serve to indicate that a more significant crisis might arise, allowing preparations to be made in advance. Our literature review revealed that this strategy is already used by peacebuilding actors (Vivekananda, 2011; Frerks, 2013), who see such events as warning signs to prepare early responses or as part of the cycle described by the ‘accordion model’. Second, relatedly, these ‘pockets’ of crisis can be seen as instances in which the development–humanitarian nexus can begin to be put into action on a relatively small and manageable scale, preparing the arrangements, negotiations among actors, and procedures to support better the transition when the crisis intensifies.

The research participants from INGOs and YNGOs underlined that this preparation and pre‐transition work would require that interventions and programmes have a certain degree of flexibility so that they can change their projects, actions, and responses. Support from organisational headquarters and donors is essential for aid actors to achieve this flexibility, which might involve temporarily combining programmes. It will also necessitate the continued availability of funding during times of crisis. As described above, the ability to achieve this transition and to allow programmes to adapt needs commitment by the donor community to continue to provide funding and to find a way for it to reach implementing actors. All of the efforts described here will entail consistent preparation and coordination work that begins well in advance of a crisis.

## Conclusion

Disasters related to hazards such as droughts, earthquakes, and floods frequently occur in conflict‐affected areas. In light of shifting levels of conflict, a large body of research and policy literature has promoted the relevance of examining how humanitarian aid and disaster response can be linked with future development and DRR initiatives, and how these actions can be better integrated throughout different conflict phases.

Yemen is a country beset by war and disaster. After more than a decade of crises and multiple development‐related programmes, Yemen saw its conflict attain such high levels of violence and intensity at the end of 2014 that a humanitarian crisis ensued. This study has analysed the subsequent transition from development to humanitarian aid in Yemen with a focus on disaster‐related actions. Special attention is accorded to water‐related hazards such as drought and water scarcity, since they constitute a severe risk in the country. Before the actual humanitarian crisis, programmes to address water problems in Yemen were part of general WASH and development initiatives, and endeavours to reduce the risk of drought were not always framed as DRR strategies. These programmes had commonly been developed in partnership with the Government of Yemen and sought long‐term solutions. Since 2015, water‐related problems have been tackled as part of the general humanitarian response, focusing on delivery by tanker trucks.

Analysing the general transition from development to humanitarian aid projects has generated important results. Notably, many international organisations (that is, INGOs, UN agencies, and other developmental organisations) scaled down their operations after the start of the crisis, and most international staff left the country. While they maintained a presence in Yemen, they did so with a reduced operating capacity, leading to the exponential growth of YNGOs, which have implemented specific aspects of the projects. Most development‐related programmes shut down, leaving Yemeni staff members unemployed or redirected to humanitarian aid activities. In the case of drought responses, this was significant, as delivering water by truck requires less staff and expertise than agriculture or sustainable livelihoods management.

Our findings also indicate that, during the HIC period, most crisis responses were carried out by YNGOs; however, many of these organisations were not well‐prepared and did not know how to lead a large‐scale humanitarian response. Although some development projects continued in a modified form, the lack of financial support from donors forced a shift to emergency aid. When development‐like aspects of projects were implemented during the HIC period, this was usually done on the initiative of local actors who had worked in development and appreciated the need to continue them. However, the expertise required to address severe droughts impeded the implementation of non‐emergency water solutions, except for a few projects in ‘pockets of development’. Identifying such needs was closely related to the notion that, in many areas of Yemen, the conflict would not directly impede interventions, making development‐related, long‐term initiatives possible. These opportunities also emerged from the development project expertise that existed in the country before the crisis.

Consequently, almost every research participant highlighted the importance of working towards better integration of pre‐crisis development projects and humanitarian aid interventions, as well as continuing to conduct as much development work as possible during the crisis. A similar idea was acknowledged by humanitarian and development actors working in Yemen at a technical meeting on 6–8 October 2015: ‘Humanitarian assistance is critical but it is not the only need. Yemen requires a broader approach that allows for support for people to cope and build resilience to recover from the crisis’ (World Bank, UNCT, and European Union, 2015, p. 13). The participants in our study asserted that this is not happening yet because conflict de‐escalation is seen as a prerequisite for development. Likewise, there are still open questions regarding what level of government stability is needed to link development, humanitarian, and disaster interventions successfully and sustainably. Without some level of peace and stability, these efforts are at risk of failure. The localisation agenda can also play a part in supporting them by linking development actors to key humanitarian sectors, better equipping them for transitioning between assistance domains and responding to needs when conflicts intensify.

Many of the challenges encountered in the transition from development relate to a lack of flexibility in programming and of space for national actors to set the agenda for interventions. YNGOs pointed to changing donor policies, a response to the escalation of conflict, as a major reason why certain development‐related activities had to be suspended, rather than limitations owing to conditions on the ground. Moreover, they signalled that the requests and suggestions that they made to donors to continue with certain activities fell on deaf ears. As a result, they had to resort to a certain level of informal, hidden action, continuing these activities within the framework of new programmes.

The findings suggest that international actors and donors involved in DRR and development should consider the continuity of their actions during times of HIC and avoid adopting an emergency mentality. Humanitarian aid actors can take a more flexible approach, building in opportunities for development‐related and DRR initiatives, even during acute emergencies, as identified by national partners

These advancements are crucial because, despite the usefulness of the humanitarian–development distinction for crisis management and analysis, ‘for those affected by crisis, the difference between humanitarian and developmental aid makes no sense’ (Gómez and Kawaguchi, 2016, p. 4).

## Acknowledgements

We would like to thank the numerous people and institutions that shared their knowledge and experiences with us. Special thanks go to Yousef Qutary, for his support during the whole research process and for feedback provided on this paper. This work was supported by the Netherlands Organisation for Scientific Research (grant number 453‐14‐013).

## Data availability statement

The data that support the findings of this study are available from the corresponding author upon reasonable request.

## Endnotes

1 HIC scenarios are usually defined as moments in protracted crises when general violence occurs and casualties owing to violent conflict surpass 1,000 (Mena, 2018).

2 Politician and Minister for Development Cooperation (1989–98) in the Netherlands.

3 Catholic Organisation for Relief and Development Aid. For more information, see https://www.cordaid.org/en/ (last accessed on 18 July 2022).

4 Initially described inadvertently as the ‘harmonica model’ (Hilhorst and Pereboom, 2015, p. 33).

5 Calculated by the authors using the Organisation for Economic Co‐operation and Development's Query Wizard for International Development Statistics (OECD, 2021). The calculation compared all commitment flows of the five categories of ODA donors (official donors, total DAC (Development Assistance Committee) countries, total non‐DAC countries, total multilateral agencies, and total private donors) to all recipients and *all sectors*, for the years 2016, 2017, 2018, and 2019, with the commitment flows of the five categories of ODA donors, to all recipients for the ‘disaster prevention and preparedness’ sub‐sector, for the years 2016, 2017, 2018, and 2019.

6 Ethical considerations were taken into account, and ethical clearance obtained. See Mena and Hilhorst (2021b) for a reflection on the ethical considerations and strategies involved when researching disasters in conflict‐affected areas.

7 For more information, see https://www.emdat.be/ (last accessed on 19 July 2022).

8 These data do not represent the International Red Cross and Red Crescent Movement, Médecins Sans Frontières, or development‐related actors such as the World Bank.

9 For more information, see https://sanaa.sites.unicnetwork.org (last accessed on 19 July 2022).

10 These reports provide information on organisational presence and activities in a particular place. Information is usually presented by cluster and type of actor: INGO, national NGO, UN, and government.

11 The Country‐based Pooled Funds system is a mechanism that allows ‘donors to pool their contributions into single, unearmarked funds to support local humanitarian efforts. This enables humanitarian partners in crisis‐affected countries to deliver timely, coordinated and principled assistance’ (OCHA, 2019c, p. 1).
